# 

**Published:** 1975-04

**Authors:** 


### Contents

The Malcolm Campbell Memorial Meeting ... 19

Obituary ... ... ... ... ... 21

The Hereditary Optic Atrophies ... ... 23

Professor W. S. Foulds

Histochemical Fibre Types in Human Extra-ocular Muscle ... ... ... ... 27
D. G. F. Harriman

Ocular Myotonia ... ... ... ... 31

Bryan Ashworth

Tables of Diagnostic Pupillary Drug Tests ... 37
H. Stanley Thompson

Paralysis of Down-gaze ... ... ... 39

David G. Cogan

Adie's Syndrome ... ... ... ... 41

Vincent Marmion

University of Bristol Examination Results ... 43

The Medical Library ... ... ... 44

Printed by F. Bailey & Son Ltd., Dursley, Glos.

